# Trabecular Bone Score in Assessing Bone Mineralization Status in Children with End- Stage Renal Disease: A Promising Tool

**DOI:** 10.1007/s00431-023-05157-z

**Published:** 2023-08-23

**Authors:** Nanees Salem, Ashraf Bakr, Riham Eid

**Affiliations:** 1 Pediatric Endocrinology and Diabetes Unit, Department of Pediatrics, Faculty of Medicine, Mansoura University, Mansoura, Egypt; 2 Pediatric Nephrology Unit, Department of Pediatrics, Faculty of Medicine, Mansoura University, Mansoura, Egypt

**Keywords:** Bone mineral density, Chronic kidney disease, DXA, Hemodialysis, Trabecular bone score

## Abstract

Areal-bone mineral density (aBMD) of lumbar-spine dual energy X-ray absorptiometry (DXA) scan is the most frequently used tool in evaluating BMD in pediatric patients, however its size dependency have significant impact on measurements accuracy in children with chronic kidney disease (CKD). This study aimed to evaluate the usefulness of trabecular bone score (TBS) computed during lumbar-spine DXA scan in assessing bone status in children on maintenance hemodialysis (HD). Ninety-three children on HD (aged 9–18 years) were subjected to lumbar-spine DXA-scan to obtain aBMD (g/cm^2^) and TBS.

Z-scores of aBMD for chronological-age (aBMD_Z-CA_), height-age (aBMD_Z-HA_), and TBS_Z-score_ were calculated using mean and SD values of 442 healthy controls. aBMD and TBS were significantly lower in short-for-age and normal height-for-age patients compared to the corresponding values of controls (*p* < 0.05 for all). Degraded vertebral microarchitecture (TBS_Z-score_ < -2) was detected in 48% and 44% of male and female patients respectively. There were no significant differences in median TBS_Z-score_ between short-for-age and normal height-for-age HD patients in male (*p* = 0.425) and in female (*p* = 0.316) patients. TBS_Z-score_ correlated significantly with aBMD_Z-CA_ (r = 0.234; *p* = 0.024) but not with aBMD_Z-HA_ (r = 0.077; *p* = 0.462). Patients with history of fractures (5 patients only) had significantly lower TBS scores compared to those without fracture history (*p* = 0.016).

*Conclusion*: TBS is significantly reduced in children on maintenance HD and is associated with increased fracture incidence. TBS has shown to be a promising tool in assessing bone quality (trabecular microarchitecture) in children with CKD being not size-dependent as is a-BMD, for further evaluation of its potential role in therapeutic and follow-up decisions.

**Table:** 

**What is Known:** *• In children with CKD, bone demineralization starts as early as CKD stage 2, so assessment of bone health is mandatory for follow up and therapeutic decisions.* *• aBMD of lumbar-spine DXA scan is the most used tool in evaluating BMD in pediatric patients, however its size dependency have significant impact on measurements made in children with CKD.*	
**What is New:** *• TBS is significantly reduced in children on maintenance HD and associated with increased fracture incidence.* *• TBS has shown to be a promising tool in assessing bone quality (trabecular microarchitecture) in children with CKD being not size-dependent as is a-BMD.*

**What is Known:**

*• In children with CKD, bone demineralization starts as early as CKD stage 2, so assessment of bone health is mandatory for follow up and therapeutic decisions.*

*• aBMD of lumbar-spine DXA scan is the most used tool in evaluating BMD in pediatric patients, however its size dependency have significant impact on measurements made in children with CKD.*

**What is New:**

*• TBS is significantly reduced in children on maintenance HD and associated with increased fracture incidence.*

*• TBS has shown to be a promising tool in assessing bone quality (trabecular microarchitecture) in children with CKD being not size-dependent as is a-BMD.*

## Introduction

Peak bone mass (PBM) corresponds to the amount of bony tissue present at the end of skeletal maturation and is considered the determinant of bone health all through life. Various factors can impact PBM including race, gender, genetic, and disease states [1]. "Chronic kidney disease-mineral and bone disorder" (CKD-MBD) describes the triad of biochemical abnormalities, bone abnormalities and extra-skeletal calcification that happens in CKD patients. In children, bone demineralization starts as early as CKD stage 2 [2, 3].

The 2017 Kidney Disease Improving Global Outcomes (KDIGO) guidelines advocate BMD testing to assess fracture risk in CKD patients [3] considering that lumbar DXA is informative in children because vertebrae are predominantly trabecular bone, and this site is readily influenced by pathologic changes, due to the rapid bone turnover [4]. However, analysis of DXA scan results in children with growth retardation is challenging because DXA is a two-dimensional technique that does not incorporate the bone depth, therefore, DXA reports bone mass as areal-BMD (aBMD) [5]. Additionally, BMD is less predictive of fracture in dialysis patients compared to general population due to the overestimation of BMD caused by arthritic conditions, scoliosis of the lumbar spine, and the occurrence of vascular calcifications [6, 7].

To overcome the drawbacks of aBMD in children with retarded growth, The International Society of Clinical Densitometry (ISCD) pediatric official positions advocated the use of “size-adjustment techniques” [8–10]. Few studies have explored these techniques in pediatric CKD and kidney transplant recipients [11, 12] including a recent report from our center which presented volumetric BMD (vBMD) as a suitable size-adjustment approach of spinal-DXA measurements in children with end-stage renal disease (ESRD) children [13].

Spine trabecular bone score (TBS) is a new imaging tool demonstrating an indirect assessment of trabecular microarchitecture of vertebrae [14, 15]. Most studies exploring TBS were conducted in adults with few studies including patients with CKD [16–18]. Scarce studies explored TBS in pediatric populations [19–21], and no such studies involving children with CKD.

In the current study, we aimed to evaluate the clinical usefulness of TBS of lumber-spine DXA measurements in assessing bone mineralization in children on maintenance hemodialysis (HD) with specific care was given to short-for-age subgroup in comparison with large sample of healthy reference children. In addition, reference range for TBS in 442 healthy Egyptian children is presented.

## Materials and methods

A case–control observational study included children with ESRD on regular HD (aged 9–18 years), recruited from Dialysis Unit at Mansoura University Children’s Hospital, over the period of 2 years. The study protocol was reviewed and approved by the local Ethics Committee of Mansoura Faculty of Medicine-Institutional Research Board (IRB) **(R.23.01.2047)**.

## Inclusion criteria

Patients were on regular HD for at least 6 months at time of enrolment, 3 sessions per week and 3–4 h duration per session. Patients’ files were revised for the monthly records of the dialysis adequacy (Kt/v values) over the previous 6 months, and the average of six Kt/v measurements was used. The Kt/v of 1.2 was considered as the standard for dialysis adequacy [22]. All patients followed a uniform protocol for management of CKD-MBD according to international guidelines [23]. None of our patients received growth hormone therapy (due to high cost and not being covered by health insurance in our country).

## Exclusion criteria

Patients with history of other chronic illnesses or medications that may affect bone health and kidney transplant recipients were excluded from this study. None of the patients had history of vertebral fractures. Patients previously received immunosuppressive therapies were also excluded.

## Methods

### Clinical evaluation

Anthropometric measurements including height and weight were obtained immediately after HD session. Body mass index (BMI; kg/m^2^) was calculated. Height age (HA) was determined as the age at which a child’s height is the median height-for-age on the growth chart. Height Z-scores (Height_Z-score_) and BMI Z-scores adjusted for HA (BMI_Z-HA_) were calculated [25], based on Egyptian reference data for healthy children [26]. Participants were also classified into three pubertal subgroups: pre-pubertal (stage I), early mid-puberty (II-III), and late-puberty (stages IV-V) or classified as delayed/ or arrested puberty [27].

### Biochemical evaluation

Blood samples were collected just before the dialysis session (mid-week) at the time of BMD and TBS measurement. Serum levels of albumin, calcium (Ca), phosphate(P), parathormone hormone (PTH) (normal values: 10–65 ng/L) and total alkaline phosphatase were measured, corrected calcium and (Ca*P) product were calculated.

### DXA measurements

Lumbar DXA scans were performed by the same technician using bone densitometer GE-Lunar Prodigy Primo system (GE Healthcare densitometer, Madison, WI, USA) at anteroposterior lumbar spine (L1-L4). DXA scans were analyzed using pediatric software (GE enCORE, v.113), that determines BMC (g) and projected bone area (cm^2^). aBMD (g/cm^2^) is calculated by dividing BMC (g) by bone area of scanned region. Spine scan provides geometric measurements of lumbar vertebrae (L1-L4) [28].

## Measurement of lumbar spine trabecular bone score (TBS)

TBS measurements were determined by TBS iNsight Software (version 2.2; Med-Imaps, France) as a gray-level textural index through analysis of the spatial organization of pixel intensity that corresponds to the differences in X-ray absorption intensity of osteoporotic bone against normal trabecular configuration [14, 15]. Given the lack of normative data for TBS in children, sex-and age-matched TBS_Z-score_ were calculated for CKD patients based on TBS mean and SD values of control subgroups. We arbitrarily defined TBS status in CKD patients as “normal microarchitecture” if TBS_Z-score_ ≥ -1, “partially degraded microarchitecture” if TBS_Z-score_ between -1 and -2 SD and “degraded microarchitecture” if TBS_Z-score_ ≤ –2 SD.

A control group consisted of 442 healthy children and adolescents aged 7–18 years (male/female: 217/225), were recruited from the same locality. The detailed characteristics, groups and BMD assessment of the controls are detailed in our previously published work [13]. The mean and SD values of TBS for each control age-subgroups were determined **(**Table 1**)** and were used to calculate Z-scores for TBS_Z-score_ in all patients. aBMD Z-scores adjusted for HA (aBMD_Z-HA_) in short-for-age HD patients were calculated as previously described [13]. Results of Z-scores were interpreted according to ISCD guidelines in pediatric population as follow, BMD_Z-score_ ≥ -1.0 reflect “normal BMD”; BMD_Z-score_ between -1.0 and -2.0 reflect "at risk of low BMD" and BMD_Z-score_ ≤ -2.0 reflect "low BMD with increased fracture risk" [8–10].

**Table 1:** Reference values of TBS of the control group of both genders

**Age group**	**Males (n = 217)**	**Females (n = 225)**	***p*-value**
**5–7 years (n = 30/33)**	1.313 ± 0.076 (1.246–1.382)	1.359 ± 0.078 (1.329–1.391)	**0.02**
**7–9 years (n = 35/38)**	1.345 ± 0.081 (1.254–1.390)	1.360 ± 0.068 (1.327- 1.389)	0.4
**9–11 years (n = 33/30)**	1.365 ± 0.037 (1.263–1.449)	1.373 ± 0.046 (1.334–1.387)	0.45
**11–13 years (n = 31/35)**	1.365 ± 0.078 (1.282–1.451)	1.386 ± 0.065 (1.340–1.460)	0.24
**13–15 years (n = 30/28)**	1.373 ± 0.066 (1.331–1.460)	1.392 ± 0.063 (1.350–1.508)	0.25
**15–17 years (n = 28/30)**	1.409 ± 0.081 (1.373–1.505)	1.475 ± 0.081 (1.423–1.514)	**0.003**
**17–18 years (n = 30/31)**	1.486 ± 0.076 (1.409–1.577)	1.487 ± 0.101 (1.451–1.616)	0.97

Data presented as mean ± SD (minimum–maximum), *TBS* trabecular bone score. Underlined values are the statistically significant (*p< 0.05)*

### Sample size

The sample size was estimated using a software program (sample size calculator) based on the study published by Bakr [24] that gave prevalence rate of 59.1% of low aBMD in children on maintenance HD and assuming 95% confidence interval, 5% level of significance, 10% margin of error and 80% study power, so the smallest number of subjects needed in HD group was 100.

### Statistical analysis

Data were analyzed using IBM SPSS Statistics, Version 20 (SPSS Inc., Chicago, IL, USA). Categorical variables were presented as number (%). Continuous variables were tested for normality, parametric variables were presented as mean ± SD and were compared using Student t-test, while non-parametric variables were presented as median and Mann–Whitney test was used for comparison between two groups and Wilcoxon signed-rank test was used to compare repeated densitometric measurements on a single sample. The variables found to be significantly correlated were introduced in linear regression analysis using TBS as dependent variables. Statistical significance was set at *p* < 0.05.

## Results

### Participants and descriptive data

Ninety-three patients (48 male; 51.6%) were enrolled in the study. Clinical and laboratory data of the patients are summarized in Table 2. Densitometric parameters according to the stature and sex of HD patients were compared between patients and controls (Table 3) which showed TBS to be significantly lower in all patient’s subgroups compared to the corresponding control group (*p* < 0.05 for all). Likewise, analysis of densitometric parameters in short-for-age HD patients (N = 72) according to age- subgroups **(**Table 4**)** showed TBS to be significantly lower in all patient’s subgroups compared to the corresponding control group (*p* < 0.05 for all).

**Table 2:** Clinical, biochemical, and densitometric characteristics of HD patients according to gender

	Male HD (n = 48)	Female HD (n = 45)
**General Characteristics**		
Age at time of study (years)	12.89 ± 2.40	13.03 ± 2.86
Height age (year)	9.24 ± 2.02	8.77 ± 1.84
∆ CA – HA (year)	3.50 (-0.5–7.5)	4.0 (0.0–8.5)
Duration of HD (months)	18.0 (6–72)	22.0 (6–105)
Primary etiology of CKD		
CAKUT	27 (56.3)	22 (48.9)
Metabolic	11 (22.9)	9 (20)
Others	10 (20.8)	14 (31.1)
**Clinical evaluation**		
Height (cm)	132.64 ± 11.85	129.91 ± 10.96
Height Z-score	-3.10 (-5.72—0.52)	-3.57 (-7.5–0.1)
BMI (kg/m^2^)	16.27 ± 3.48	17.86 ± 3.05
BMI Z-score for HA	-0.37 (-1.5–3.8)	0.06 (-3.1–2.2)
**Biochemical evaluation**		
Calcium (mg/dL)	7.88 ± 1.12	8.16 ± 1.09
Phosphorus (mg/dL)	6.70 (3.1–13.7)	5.60 (2.7–13.1)
Calcium*Phosphorus products (mg/dL)	51.17 (27.28–96.32)	48.95 (23.22–84.05)
Alkaline phosphatase (U/L)	550.0 (143–1565)	512.44 (155–1400)
Parathyroid hormone (ng/L)	542.40 (123–3079)	465.10 (107–2924)
**Lumbar Spine (L1-L4) DXA scan**		
LS-aBMD (g/cm^2^)	0.659 ± 0.14 (0.359–0.898)	0.714 ± 0.11 (0.388–1.018)
LS-aBMD Z-score for CA	-2.52 (-4.50–1.37)	-2.78 (-5.31–0.79)
LS-aBMD Z-score for HA	-1.58 (-2.97–2.25)	-1.69 (-3.20–1.92)
LS-TBS	1.319 (1.032–1.423)	1.309 (0.863–1.468)
LS-TBS Z-score	-1.31 (-3.75 – 1.23)	-1.22 (-3.02–1.27)

Data presented as number (%), mean ± SD or median (minimum–maximum)

*aBMD* areal bone mineral density, *BMI* body mass index, *CA* chronological age, *CAKUT* congenital anomalies of kidney and urinary tract, *CKD* chronic kidney disease, *DXA* dual energy X-ray absorptiometry, *eGFR* estimated glomerular filtration rate, *HA* Height age, *HD* hemodialysis, *TBS* trabecular bone score, *vBMD* volumetric bone mineral density

**Table 3:** Densitometric parameters according to the stature of patients compared to age-and sex-matched controls

	**Males**					
	**Short-for-age patients** **(n = 37)**	**Age-matched control** **(n = 152)**	***P*- value**	**Normal height-for-age HD patient** **(n = 11)**	**Age-matched Control** **(n = 64)**	***P*- value**
**Age (year)**	12.71 ± 2.50	13.36 ± 3.45	0.468	10.92 ± 1.23	11.09 ± 0.92	0.606
**aBMD (g/cm**^**2**^**)**	0.649 ± 0.135	0.812 ± 0.139	** < 0.001**	0.689 ± 0.148	0.765 ± 0.093	**0.034**
**TBS**	1.299 ± 0.097	1.401 ± 0.066	** < 0.001**	1.330 ± 0.091	1.369 ± 0.045	**0.013**

	**Females**					
	**Short-for-age patients** **(n = 35)**	**Age-matched control** **(n = 154)**	***P*- value**	**Normal height-for-age patients** **(n = 10)**	**Age-matched female control** **(n = 93)**	***P*- value**
**Age (year)**	13.02 ± 2.96	13.68 ± 2.73	0.238	10.80 ± 2.15	11.56 ± 1.90	0.277
**aBMD (g/cm**^**2**^**)**	0.613 ± 0.14	0.871 ± 0.18	** < 0.001**	0.693 ± 0.13	0.789 ± 0.13	**0.047**
**TBS**	1.288 ± 0.13	1.405 ± 0.07	** < 0.001**	1.313 ± 0.10	1.383 ± 0.05	**0.005**

Data presented as mean ± SD. Underlined values are the statistically significant (*p* < 0.05)

*aBMD* areal bone mineral density, *HD* hemodialysis, *TBS* trabecular bone score, *vBMD* volumetric bone mineral density

**Table 4:** Analysis of densitometric parameters according to age-subgroups of patients compared to matched controls

	**Males**			**Females**		
	**Short HD patient** **(n = 37)**	**Age-matched** **control** **(n = 152)**	***P*- value**	**Short HD patient** **(n = 35)**	**Age-matched** **control** **(n = 154)**	***P*- value**
**Group 1 (9–11 years)**						
Number	n = 11	n = 33		n = 10	n = 30	
aBMD (g/cm^2^)	0.636 ± 0.17	0.748 ± 0.08	**0.024**	0.609 ± 0.12	0.753 ± 0.11	**0.002**
TBS	1.222 ± 0.10	1.365 ± 0.04	** < 0.001**	1.249 ± 0.14	1.373 ± 0.05	** < 0.001**
**Group 2 (11–14 years)**						
Number	n = 14	n = 52		n = 12	n = 54	
aBMD (g/cm^2^)	0.588 ± 0.11	0.879 ± 0.12	** < 0.001**	0.735 ± 0.14	0.861 ± 0.15	**0.025**
TBS	1.316 ± 0.09	1.416 ± 0.06	** < 0.001**	1.345 ± 0.10	1.429 ± 0.05	**0.030**
**Group 3 (14–18 years)**						
Number	n = 12	n = 67		n = 13	n = 70	
aBMD (g/cm^2^)	0.736 ± 0.08	1.067 ± 0.13	** < 0.001**	0.771 ± 0.12	1.099 ± 0.10	** < 0.001**
TBS	1.351 ± 0.05	1.487 ± 0.06	< **0.001**	1.233 ± 0.08	1.503 ± 0.08	** < 0.001**

Data presented as mean ± SD. * Significant difference (P < 0.05)

*aBMD* areal bone mineral density, *HD* hemodialysis, *TBS* trabecular bone score, *vBMD* volumetric bone mineral density

## Main results

There were no significant differences in median Z-scores of TBS between short-for-age and normal height-for-age HD patients in male HD patients (-1.36 (-3.75–1.10) vs. -1.28 (-2.85–1.23); *p* = 0.425) and in female HD patients [-1.17 (-3.02 -1.27) vs. -1.05 (-2.41- 0.95); *P* = 0.316]. Additionally, no significant difference detected in median TBS_Z-score_ aBMD_Z-CA_ and aBMD_Z-HA_ between 3 primary etiological groups (congenital anomalies of kidney and urinary tract (CAKUT), metabolic, others), *p* = 0.055, 0.8 and 0.2 respectively.

Correlation analysis between clinical, biochemical, and densitometric parameters among HD patients to aBMD and TBS _Z-score_ are presented in Table 5.

**Table 5:** Correlation analysis between clinical, biochemical, and densitometric parameters among HD patients

		aBMD_Z-CA_	aBMD_Z-HA_	TBS_Z-score_
**Age (year)**	r	-0.406	0.187	-0.313
	*p*	< 0.001*	0.073	0.002*
**Height**_**Z-score**_	r	0.395	-0.253	0.134
	*p*	< 0.001*	0.014*	0.199
**BMI**_**Z-HA**_	r	0.236	0.333	0.146
	*p*	0.046*	0.004*	0.061
Ca*P products (mg/dL)	r	-0.327	-0.138	**-0.521**
	*p*	0.001*	0.188	< 0.001*
**Alkaline phosphatase (U/L)**	r	0.148	0.075	0.064
	*p*	0.156	0.474	0.539
**Parathyroid hormone (ng/L)**	r	-0.154	0.097	-0.058
	*p*	0.139	0.357	0.583

*aBMD* areal bone mineral density, *BMI* body mass index, *CA* chronological age, *HA* height age, *HD* hemodialysis, *TBS* trabecular bone score

*Significant correlation (*p* < 0.05)

### Distribution of HD patients according to Z-score of densitometric parameters

#### Distribution of male HD patients (Fig. 1a)

**Fig. 1:**
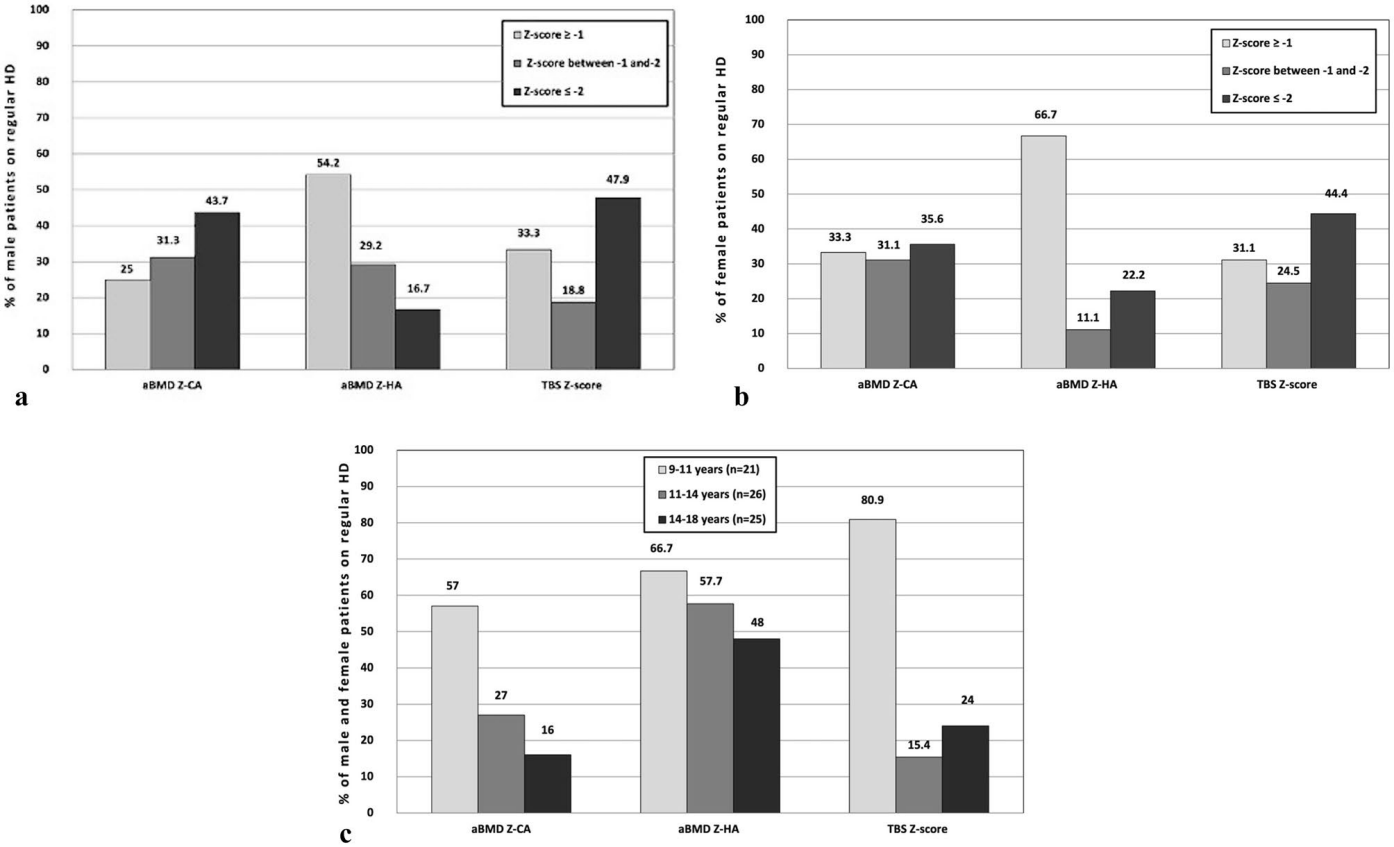
(**a**) Distribution of male patients according to Z-score of densitometric parameters, (**b**) distribution of female patients according to Z-score of densitometric parameters, and (**c**) distribution of male and female patients with densitometric parameters Z-score of ≥ 1 according to age subgroups.  aBMD, areal bone mineral density; CA, chronological age; HA, height age; TBS, trabecular bone score

Regarding aBMD_Z-CA_, 43.7% of male patients had Z-score ≤ –2, whereas 25% of male patients had a normal Z-score ≥ -1. Regarding aBMD_Z-HA_, 54.2% of male patients had normal Z-scores ≥ -1, while 16.7% of male patients had Z-scores ≤ -2 for aBMD_Z-HA_. With respect to TBS_Z-score_, 47.9% male patients had degraded vertebral microarchitecture.

#### Distribution of female HD patients (Fig. 1b)

Regarding aBMD_Z-CA_, nearly equal distribution of female patients among the three Z-score groups where 33.3% of female patients had a normal Z-score ≥ -1, and 35.6% of female patients had Z-score ≤ –2 while 22.2% of female patients had Z-scores ≤ –2 for aBMD_Z-HA_. With respect to TBSZ-score, 44.4% female patients had degraded vertebral microarchitecture.

#### Distribution of male and female HD patients with densitometric parameters Z-Scores ≥ -1 based on age-subgroups (Fig. 1c)

The percentage of normal aBMD_Z-CA_ and aBMD_Z-HA_ were almost similar at younger age group (9–11 years) (57% and 66.7% respectively) with nearly equal proportion. At 11–14 years-old group, greater discordances were detected between the percentage of HD patients with normal aBMD_Z-HA_ (57.7%) and aBMD_Z-CA_ (27%) with a proportion of 1:2. The highest discordance was observed in the older age group (14–18 years-old) between the percentage of aBMD_Z-HA_ (48%) and aBMD_Z-CA_ (16%) with a proportion of 1:3.With respect to TBS_Z-score_, the group of HD patients with normal TBS_Z-score_ was higher (80.9%) in younger age group (9–11 years) and decreased in patients older than 11 years.

### Correlation analysis between clinical, biochemical, and densitometric parameters in HD patients

Among total HD patients, aBMD_Z-CA_ and TBS_Z-score_ correlated negatively with age (r = -0.406; *p* < 0.001 and r = -0.313; *p* = 0.002 respectively). BMD_Z-CA_ correlated positively with height_Z-score_ (r = 0.0.395; *p* < 0.001) and BMI_Z-HA_ (r = 0.236; *p* = 0.046). aBMD_Z-HA_ correlated negatively with height_Z-score_ (r = -0.253; *p* = 0.014) and positively with BMI_Z-HA_ (r = 0.333; *p* = 0.004), but not correlated with age. TBS_Z-score_ does not have a significant correlation with height_Z-score_ (r = 0.134; *p* = 0.199) or BMI_Z-HA_ (r = 0.146; *p* = 0.061). Although the correlation between the clinical, biochemical, and densitometric parameters is significant, it is of low magnitude.

In addition, negative correlations were detected between Ca*P product and both BMD_Z-CA_ (r = -0.327; *p* = 0.001) and TBS_Z-score_ (r = -0.521; *p* < 0.001) but not with BMD_Z-HA_.

None of densitometric parameters displayed significant association with the duration of HD, the measurements of dialysis adequacy (Kt/v), or serum alkaline phosphatase and PTH levels.

### Correlation analysis between Z-scores of densitometric parameters in HD patients

**Fig. 2:**
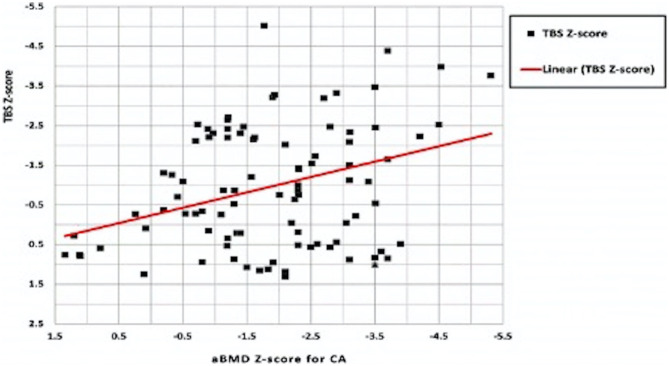
Correlation analysis between Z-scores of lumbar spines densitometric parameters in HD patients. aBMD, areal bone mineral density; HD, hemodialysis; TBS, trabecular bone score

aBMD_Z-CA_ was shown to correlate significantly with TBS_Z-score_ (r = 0.234; *p* = 0.024) **(**Fig. 2**)** while aBMD_Z-HA_ didn’t correlate with TBS_Z-score_ (r = 0.077; *p* = 0.462).

Excluding normal height-for-age HD patients (n = 21) from correlation analysis had a negligible effect on the previous correlation results.

Patients with history of fractures (5 patients) had significantly lower TBS scores compared to those without fracture history [(*p* = 0.02) (TBS 1.12 ± 0.05 versus 1.45 ± 0.3)], while BMD did not show significant difference between patients with and without fracture history.

## Discussion

Bone biopsy is considered the gold standard for bone status assessment in CKD patients [23], but it is expensive, invasive and demands expertise in the interpretation of bone pathology. On the other hand, currently available non-invasive measures, including DXA and biomarkers of bone formation/resorption, are affected by growth and pubertal status and have limited sensitivity and specificity in predicting changes in bone turnover and mineralization [29].

Marked discrepancies exist in the results of previous reports on DXA-derived BMD in children with CKD [30–37] which emphasizes the urging need for a measure or size-adjustment techniques to make DXA-derived bone mineralization assessment more reliable in assessing bone status in children with impaired growth including those with ESRD. In our previous work [13] we explored size-adjustment approaches to lumber spine DXA measurements concerning height age-adjusted BMD _Z-scores_ and estimated vBMD as potential tools in BMD assessment in CKD children.

In the current work, we evaluated for the first time (to the best of our knowledge) the usefulness of DXA-derived TBS in bone mineralization status assessment in children on maintenance HD compared to large sample of healthy controls. TBS has recently been used in studies involving pediatric populations in health states [18–20] and to examine effects of diseases as anorexia nervosa, cerebral palsy (CP), inflammatory bowel diseases, neurofibromatosis and diabetes [38–42].

Frailty is a condition, initially recognized in elderly populations, characterized by a decrease in resistance to minor stress actions due to reduced biological reserves. In adult CKD, anorexia and reduced physical activity together with the accumulation of pro-inflammatory cytokines, metabolic acidosis, and vitamin D deficiency and insulin-like growth factor-1 (IGF-1) signaling derangement, may contribute to the development of frailty [43]. Few studies in adults with CKD stated that TBS may indicate a phenotype of fragility and a CKD-MBD phenotype reciprocal to cardiovascular events [44], and that fracture prevalence considerably correlated inversely with TBS supporting the role of TBS as an added tool for fracture risk estimation in patients with ESRD [45]. The term frailty was recently associated with bone mineral apparent density in children with CKD pediatric population [43, 46] and not correlated before to TBS (to the best of our knowledge).

In the current work, analysis of densitometric data showed that, the means of aBMD, and TBS were drastically lower in short-for-age and normal-height-for-age HD patients compared to matched controls. Remarkably, the level of significance was more obvious in short than in normal-height patients, which was also observed in our earlier report concerning vBMD [13] reflecting the influence of poor growth to low BMD in addition to CKD-related risk factors that adversely affect bone accretion in such patients.

However, the analysis of densitometric findings in short-for-age patients established on age-subgroups showed that TBS was considerably lower in short-for-age patients compared to controls in all age groups which is not coherent with vBMD assessment which displayed significant difference between patients and controls in the prepubertal group [13].

Studies in healthy pediatric populations reported a steady increase in TBS with aging and maturation of TBS are achieved during late puberty, before attainment of the spinal peak bone mass by 2 or more years [19, 20]. Rehberg et al., in their cohort of CP patients also reported that TBS did not increase with age until an inflection point at 10 years in females, and 12 years in males [39]. A similar trend of TBS increase is observed in out cohort of healthy subjects with significantly higher values observed in females compared to males in age group (15–17 years) which is consistent with published reference values [47] and could be justified by earlier puberty in females. Additionally, on the forearm, boys expand their cortical bone area mainly periosteally during puberty, leading to augmented bone strength, whereas in girls endocortical apposition is a famous mechanism in puberty, believed to work as calcium reserve for childbearing [48]. These TBS values differences in relation to age group, sex and puberty reported in the present work and previous studies [19, 49] support the need for TBS age-sex adjusted reference values. The TBS values variation by ethnicity in children is not established unlike aBMD in both children and adults, which has been found to be higher in African Americans versus other ancestry groups [50, 51] while Kalkwarf et al., [47] reported no difference in TBS by African ancestry in their cohort of healthy children aged 5–20 years.

In the current study, the higher proportion of HD patients having degraded vertebral microarchitecture and prior affection of TBS compared to other BMD Z-scores, strengthening the ideas that the TBS assesses diverse components of bone health, and reflects the earlier deleterious effect of CKD on trabecular bone microarchitecture that become more prominent with aging due to the contribution of pubertal delay/arrest rather than the burden of short stature. Our assumption is supported by the lack of significant differences in TBS_Z-score_ with respect to the stature of HD patients, and TBS_Z-score_ was negatively correlated with age but not correlated with height_Z-scores_. These findings are consistent with a study in a cohort of CP patients which also reported that TBS was not influenced by age-adjusted height Z-scores, mobility levels or body composition giving the use of TBS a privilege over aBMD when assessing bone health [39].

The results of correlation analysis revealed significant negative correlations between Ca*P product and both BMD_Z-CA_ and TBS_Z-score_ but not with BMD_Z-HA_. In addition to a significant correlation between TBS_Z-score_ and BMD_Z-CA_ but not with BMD_Z-HA_. These observations could be related to that the correction of BMD for HA overestimates its value and thus possibly disturb its correlations to bone minerals and TBS values. By convention, the use of HA approach as an adjustment technique do not take into account the patients´ age, thus older short-for-age HD patients were compared with HA-matched controls who are younger, and at an earlier stage of sexual maturation [5, 52].

Similar to prior studies, children with fracture history had significantly lower TBS compared to those without previous fracture events. There are several reasons for the increased risk of fracture in dialysis patients, besides renal osteodystrophy; sarcopenia, disability, malnutrition, autonomic dysfunction and neuropathy in HD patients increase their risk of falling [16, 44].

## Strengths and limitations

The current study is the first to assess the possible role of TBS in evaluating the bone status in a homogenous relatively representative sample of children with ESRD in comparison to large sample of healthy children.

The limitations of the study are being a single center, cross-sectional study and not studying the influence of skeletal maturity, puberty, lean body mass, physical activity, fatigue, and medications on DXA measurements. Additionally, vitamin D status and fibroblast growth factor 23 (FGF-23) were not assessed and the limited number of patients with fracture history, although totally justified by the rarity of this condition in pediatric population, limits the strength of the results. Therefore, there is a need for further large-scale multicenter studies to confirm the possible utility of TBS for predicting the risk of fracture and to correlate TBS values to vitamin D and FGF-23 levels in this vulnerable population.

## Conclusion

Spinal TBS is a promising tool in BMD evaluation in pediatric CKD for two reasons; first, TBS was not correlated with height, and second, pubertal delay with sex steroids deficiencies that commonly exist in CKD, is associated with microarchitecture deterioration with increased fracture risk at sites rich in trabecular bone as vertebral bodies. Pediatric reference data for TBS for different ethnic groups are also crucial for generation of age-, sex-, and race-specific Z-scores. Further longitudinal studies looking at the predictive values of TBS on fracture and cardiovascular events are needed to improve management of HD children.

## Data Availability

Relevant, de-identified data can be made available on request.

## Abbreviations

**aBMD**: Areal-bone mineral density
**BMC**: Bone mineral content
BMI: Body mass index
**CA**: Chronological age
**CKD-MBD**: Chronic kidney disease-mineral and bone disorder
**DXA**: Dual energy X-ray absorptiometry
**ESRD**: End stage renal disease
**HA**: Height age
**HD**: Hemodialysis
**MRI**: Magnetic resonance image
**ISCD**: International Society of Clinical Densitometry
**CAKUT**: Congenital anomalies of kidney and urinary tract
**KDIGO**: Kidney Disease Improving Global Outcomes
**PTH**: Parathyroid hormone
**PBM**: Peak bone mass
**TBS**: Trabecular bone score
**vBMD**: Volumetric-bone mineral density

## Declarations

### Permission note

All materials in the manuscript are original.
